# Leukocyte Apheresis in the Management of Ulcerative Colitis

**DOI:** 10.4103/1319-3767.56093

**Published:** 2009-10

**Authors:** Ahmed Helmy, Maheeba Abdulla, Ingvar Kagevi, Khalid Al Kahtani

**Affiliations:** Section of Gastroenterology, Department of Medicine, King Faisal Specialist Hospital & Research Centre, Riyadh, Saudi Arabia

**Keywords:** Cytapheresis, granulocytapheresis, therapy, inflammatory bowel disease, induction of remission

## Abstract

Ulcerative colitis is a chronic inflammatory disease that affects the colon and rectum. Its pathogenesis is probably multifactorial including the influx of certain cytokines into the colonic mucosa, causing disease activity and relapse. The hypothesis of removing such cytokines from the circulation by leukocytapheresis was implemented to reduce disease activity, maintain remission, and prevent relapse. Many recent reports not only in Japan, but also in the West, have highlighted its beneficial effects in both adult and pediatric patients. Large placebo-controlled studies are needed to confirm the available data in this regard. In this article, we shed some light on the use of leukocyte apheresis in the management of autoimmune diseases, especially ulcerative colitis.

Ulcerative colitis (UC) is a chronic inflammatory bowel disease (IBD) with a relapsing and remitting course. It affects many individuals worldwide with deleterious effects on the quality of life.[1,2] Many medications, including anti-inflammatory drugs, corticosteroids, immunosuppressive drugs, and biological agents, are used to induce and maintain remission without curing the disease and with many side effects.[3,4]

The pathogenesis of UC is ill-understood, and seems to result from a complex interplay between susceptibility genes, environmental factors, and the immune system. Many inflammatory cytokines, such as tumor necrosis factor (TNF)-α, interleukin (IL)-1β, IL-6, IL-8, and others are involved.[5,6] The sources of these cytokines are the activated peripheral blood granulocytes which get mobilized first, then infiltrate the colonic mucosa and interact with lymphocytes to orchestrate the inflammatory response and initiate disease activity and/or relapse.[7–10] Therefore, removal of these activated granulocytes by extracorporeal cytapheresis systems, *i.e*., leukocytapheresis and granulocytapheresis may be a logical therapeutic maneuver.

The goal of this concise report is to present the available data on the efficacy, safety, and applicability of cytapheresis in patients with UC.

## DEFINITION

Cytapheresis is an extracorporeal removal of specific cells from the blood using special filters or columns. Due to its ability to remove white blood cells, cytapheresis has been used as a therapeutic modality in many diseases in which sensitized white cells have a pathogenic effect.[11,12] Leukocytapheresis and granulocytapheresis were mainly used in Japan, but over the last decade, they have also attracted much attention in Europe and North America.

## SYSTEMS

**Figure 1:**
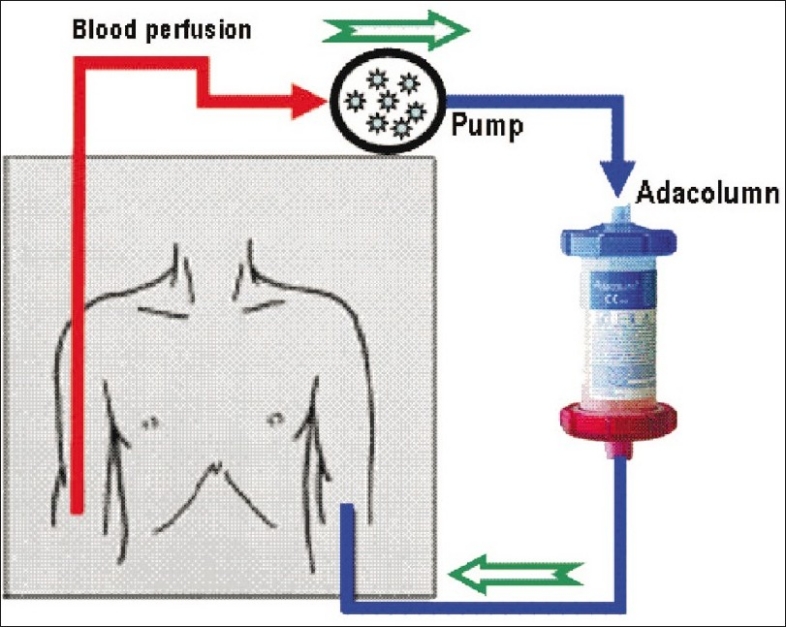
Schematic diagram of the leukocyte apheresis system

Cellsorba (Asahi Medical, Tokyo, Japan) and Adcolumn (Japan Research Laboratories, Takasaki, Japan, Figure 1) are the commonly used aphesesis systems in the current literature. The whole venous blood is perfused through an adsorption column. The blood is pumped from a peripheral vein in one arm, filtered, and returned to the body via the other arm. A total of 1800 mL blood is filtered over a period of 60 min. The device which uses an Adacolumn filter is preferred over the Cellsorba filter as it selectively removes activated granulocytes and monocytes with no significant change in the number of lymphocytes or platelets.[13]

## CLINICAL USES

The beneficial effect of Adacolumn is related to its ability to reduce numbers of granulocytes, monocytes, and inflammatory cytokine levels (TNF-α, IL-1β, IL-6, and IL-8). Additionally, the Adacolumn increases the concentration of circulating immature neutrophils and reduces their ability to secrete such pro-inflammatory cytokines.[14–16] Cytapheresis was first used in Japan for treating patients with leukemia in the 1980s, and is currently used in several autoimmune diseases including rheumatoid arthritis, systemic lupus erythematosus, psoriasis, and IBD with varying degrees of success [Table 1]. In UC, cytapheresis is indicated in patients with moderately severe cases (steroid-resistant or naïve cases), intractable disease (steroid-dependent), and in severe or fulminant disease.[17]

**Table 1:** Common clinical applications* of leukocytapheresis/granulocytapheresis

Ulcerative colitis
Rheumatoid arthritis
Behcet's disease
Systemic lupus erythematosus
ANCA-associated vasculitis
Anaphylactoid purpura
Primary biliary cirrhosis
Crohn's disease
Chronic hepatitis C
Carcinomas
Others

* The list is not exhaustive

## ADVANTAGES

The principle of cytapheresis is to remove badly programmed cells instead of adding medications; this explains its relative safety. Cytapheresis does not require shunt operation, as in chronic hemodialysis, does not exacerbate anemia, nor does it influence hemodynamic parameters.[18,19] Each apheresis session lasts for 60 min and can be done in an outpatient setting. Also, it has good tolerability as each treatment course for UC consists of a single session per week for five weeks. In addition, it may improve patients' quality of life.[2] It should be noted that cost-effectiveness has not been studied in this type of patients. Leukopheresis for IBD has not been recommended by US-FDA as first-line or even second-line treatment until now.

## DISADVANTAGES

Most of the adverse effects are mild and transient and are attributed to the extracorporeal circulation. These include dizziness, headache, fever, chills, nausea, vomiting, and abdominal pain. In addition, apheresis is a costly procedure compared to other therapeutic modalities. However, it is cost-effective as it reduces the number of hospital admissions, the treatment for steroid-induced side-effects, the need for the expensive biological therapies, and/or the need for surgery.[17]

## EFFICACY IN UC

Many studies have been conducted to evaluate the efficacy of cytapheresis in the induction of remission in patients with IBD [Table 2].[19–42] Most of these studies were conducted in small numbers of patients with variable disease severity, and were open-label, uncontrolled studies; a few were randomized.[22,25,33] However, they all supported the effectiveness of cytapheresis in reducing disease activity, achieving clinical remission, and enhancing mucosal healing.

**Table 2:** Summary of studies using cytapheresis in treating patients with ulcerative colitis

Reference	#	UC disease status	Cases #	Apheresis protocol	Side effects %	Efficacy %
Shimoyama *et al.* 2001	29	Refractory to conventional drugs	53	Standard*	9	21
Tomomasa *et al.* 2003	28	Steroid-refractory	12	Once weekly for 5–10 weeks	9	67
Hanai *et al.* 2003	30	Steroid-dependent	31 & 8	10-11 sessions in 11 weeks	18	81-88
Suzuki *et al.* 2004	31	Steroid-naive	20	Twice weekly for 3–5 weeks	10	85
Naganuma *et al.* 2004	32	Steroid-dependent and-refractory	44	Standard*	5	55
Hanai 2004**	33	Steroid-dependent	46	11 sessions in 10 weeks	22	83
Yamamoto *et al.* 2004	34	Mild-to-moderate and distal	30	Standard*	27	70
Domenech *et al.* 2004	35	Steroid-dependent	14	Standard*	15	62
Kanke *et al.* 2004	36	Mild-moderate	60	10 sessions in 12 weeks	18	23
Kim *et al.* 2005	37	Refractory to conventional drugs	27	Standard*	11	70
Sawada *et al.* 2005	38	Moderate-severe	10	Standard*plus 2 more sessions in 4 weeks	10	80
Kruis *et al.* 2005	39	Steroid-dependent	35	Standard*	3	37
D'Ovidio *et al.* 2006	40	Mild-moderate dependent/refractory	12	Standard*	0	25$
Ikeda *et al.* 2006	26	Moderate-severe	4	Standard*	-	50
Sands *et al.* 2006	41	Moderate-severe	15	Standard*	0	33
Okada *et al.* 2006	24	Moderate-severe	6	Once per week for 4 weeks	17	83
Kumagai *et al.* 2007	27	Recurrent (*n* = 4) and first attack (*n* = 1)	5	Standard* but at a rate of 50 mL/min	20	60
Bresci *et al.* 2007	21	Acute	20	Standard*	10	70
Takemoto *et al.* 2007	23	Steroid-refractory	71	1–2 sessions / week for 2–10 weeks	-	75##
Emmrich *et al.* 2007	20	Refractory to conventional drugs	20	Standard* plus 1 session/month for 6 months	-	70
Ljung *et al.* 2007	19	Steroid-dependent	52	Standard*	15	48
Aoki *et al.* 2007	42	Moderate-severe	22	2–3 sessions / week, total up to 10	-	75
Sakuraba *et al.* 2008**	25	Moderate	30	Standard* (*n* = 15) vs. Intensive (*n* = 15)		66.7 *vs.* 80
Hanai *et al.* 2008**	22	Moderate-severe U	35	Twice weekly × 3 then once up to 11 sessions	14	73.4

* Standard protocol = 1 session per week for five consecutive weeks, each for 60 min, with blood flow rate of 30 mL/min.

** These studies are randomized controlled trial apheresis *versus* prednisolone. vs. = Versus,

# = number,

$ = Improvement assessed endoscopically,

## = in this study only 19 (27%) patients maintained remission for more than 6 months.

Interestingly, cytapheresis in pediatric patients has similar effectiveness in inducing as well as maintaining remission in steroid refractory UC as in adults. This can reduce the serious steroid-induced complications such as growth retardation, infection, and cosmetic effects.[26–28]

## REGIMENS

Standard (conventional) course: One session per week for five consecutive weeks.

Intensive course: 2–3 sessions per week in the first two weeks, then once weekly. An intensive cytapheresis course induces rapid remission and is, therefore, a preferred regimen compared to the standard once-weekly course.[25,42]

## POTENTIAL ROLE OF PHOTOPHERESIS

Extracorporeal photopheresis (ECP) is the *ex vivo* exposure of apheresed peripheral blood mononuclear cells to ultraviolet A light in the presence of a DNA-intercalating agent such as 8-methoxythoralin (8-MOP), and their subsequent reinfusion. ECP was used initially since the early 1980s in managing malignant and autoimmune diseases including Sezary syndrome, T-cell lymphoma, and graft-versus-host disease.[43–46] In ECP, exposure of circulating immune cells to UVA and 8-MOP induces immunomodulatory changes that lead to tolerance to alloreactive or autoreactive antigen-generated T-cell responses.[47,48] The potential role of ECP in combination with apheresis in patients with IBD has not been tested, and warrants further investigation.

## CONCLUSIONS

Cytapheresis may offer an adjuvant therapeutic option for inducing and maintaining remission in patients with chronic active UC. It is associated with a low incidence of adverse effects compared to other modalities. Well-designed placebo-controlled trials as definitive proofs of efficacy are currently underway. Also needed are studies addressing optimal treatment schemes, patients who would benefit most from this modality, when to combine it with other therapies such as immunotherapy, and the value of using ECP.
